# Unsupervised Global CBC/RUO/CPD Phenotyping Identifies Haematological Clusters Enriched for Thrombocytopenia Severity and Mechanisms

**DOI:** 10.1111/ijlh.70217

**Published:** 2026-08-11

**Authors:** Mohammad A. Altememi, Emmanuel J. Favaloro, Md Zahidul Islam, Sara Ng, Danny Hsu, Abishek B. Santhakumar

**Affiliations:** ^1^ School of Dentistry and Medical Sciences, Faculty of Science and Health Charles Sturt University Wagga Wagga New South Wales Australia; ^2^ Haematology Laboratory Liverpool Hospital Liverpool New South Wales Australia; ^3^ Sydney Centres for Thrombosis and Haemostasis, Institute of Clinical Pathology and Medical Research (ICPMR) and Research and Education Network (REN) Westmead Hospital Westmead New South Wales Australia; ^4^ AI and Cyber Futures Centre Charles Sturt University Bathurst New South Wales Australia; ^5^ School of Computing, Mathematics and Engineering Charles Sturt University Bathurst New South Wales Australia; ^6^ Department of Haematology, Liverpool Hospital, Faculty of Medicine & Health University of New South Kensington New South Wales Australia

**Keywords:** cell population data, clustering, complete blood count, thrombocytopenia, unsupervised machine learning

## Abstract

**Introduction:**

Thrombocytopenia has heterogeneous mechanisms, including peripheral destruction, consumption, and reduced marrow production. This study developed an unsupervised global complete blood count/research‐use‐only/cell population data (CBC/RUO/CPD) phenotyping model and evaluated post hoc whether thrombocytopenia severity and clinically adjudicated mechanisms were enriched within the resulting clusters.

**Methods:**

Retrospective haematology analyser data from 254 289 unique specimens were analysed after cleaning 537 279 extracted rows. Exploratory missingness filtering retained 123 features and 173 851 complete‐case specimens. The final model used a refined 41‐feature CBC_EXTENDED complete‐case dataset containing 241 591 specimens. Features were standardised, reduced using principal component analysis to 20 components explaining 92.94% of cumulative variance, and clustered using k‐means. Thrombocytopenia thresholds and electronic health record (EHR) adjudicated primary mechanism labels were evaluated post hoc.

**Results:**

The final global k‐means model (k = 4) generated four broad haematological clusters comprising 14%–37% of specimens. Internal separation was modest but interpretable, with sampled silhouette 0.10, Davies–Bouldin 2.21, and Calinski–Harabasz 6536.92 in the fixed 60 000 specimen model comparison analysis. Cluster 3 showed progressive post hoc enrichment with thrombocytopenia severity, accounting for approximately 46%, 60%, and 71% of specimens with platelet counts < 150, < 100, and < 50 × 10^9^/L, respectively. In 225 clinically annotated thrombocytopenic specimens, k‐means k = 4 showed limited but non‐random concordance with adjudicated mechanisms (purity 0.59; adjusted Rand index 0.14; normalised mutual information 0.18).

**Conclusion:**

Unsupervised global CBC/RUO/CPD clustering identified broad laboratory phenotypes with post hoc enrichment for thrombocytopenia severity and adjudicated mechanisms. These findings support underutilised analyser‐derived parameters for laboratory phenotyping and hypothesis generation, not standalone diagnosis.

## 1 Introduction

Thrombocytopenia is one of the most common hematologic abnormalities [1], usually defined as platelet counts of < 150 × 10^9/L. Thrombocytopenia is often discovered incidentally during routine complete blood count (CBC) testing. The etiology of thrombocytopenia is not usually obvious and so additional, time consuming, potentially invasive and often costly investigations are required. Notably, thrombocytopenia is sometimes a first sign of severe and concerning scenarios such as hematologic malignancies, infectious diseases, thrombotic microangiopathies, and autoimmune disorders, and is also a common side effect of many medications. Indeed, more than 200 diseases include low number of platelets among their symptoms [1]. Significantly reduced platelet counts may also cause spontaneous bleeding and may require emergency hematology consultation and management. Patients who present with thrombocytopenia as part of a multisystem disorder are usually ill and require urgent evaluation and treatment [2]. Additional life‐threatening causes of thrombocytopenia encompass immune thrombocytopenic purpura (ITP), heparin‐induced thrombocytopenia (HIT), liver disease, thrombotic thrombocytopenic purpura (TTP)/haemolytic uremic syndrome (HUS), and disseminated intravascular coagulation (DIC). During pregnancy, preeclampsia and the HELLP (haemolysis, elevated liver enzymes, and low platelet count) syndrome/features are also associated with thrombocytopenia, as is gestational thrombocytopenia [2]. Treatment of thrombocytopenia is based on the etiology and, in some cases, treating the primary cause results in normalisation of platelet counts [2].

Broadly speaking, causes of thrombocytopenia can be divided into two broad mechanisms, either underproductive (i.e., reduced production of platelets by the bone marrow) or overdestructive (i.e., increased consumption of platelets through peripheral destruction) [1, 2]. Underproductive thrombocytopenia is associated with bone marrow failures (e.g., aplastic anaemia, leukemia, myelodysplasia, chemotherapy effects), which often concurrently cause anemia and leukopenia [1]. Overdestructive thrombocytopenia encompasses immune‐mediated platelet destruction (such as ITP, HIT) and consumptive processes (e.g., DIC, sepsis‐associated thrombocytopenia), where peripheral platelet destruction or utilisation is accelerated [1].

Distinguishing between these etiologies is clinically important because it vastly changes management (e.g., immunosuppression for ITP vs. treating infection vs. supportive transfusion in hypoplastic states). However, this distinction can often be challenging due to the heterogeneity of causes of thrombocytopenia. Patients with similar platelet counts can have very different pathologies and traditional diagnostic algorithms rely primarily on routine CBC, clinical manifestations and invasive tests such as bone marrow biopsy, rather than CBC alone [3]. With rapidly developing detection and sensor technologies, advanced automated hematology analysers can provide additional pieces of information in conjunction with the CBC regarding cell morphology without additional costs or patient burden. For example, the Beckman Coulter DxH platform can provide measurements of cell population data (CPD) and research use only (RUO) items that inform of different peripheral blood (PB) cells using VCS (volume, conductivity, and scattering) technology [4]. CPD items provide information about white blood cell (WBC) volume, conductivity, and light scattering from five additional angles, including median angle light scatter (MALS), upper median angle light scatter (UMALS), lower median angle light scatter (LMALS), low angle light scatter (LALS), and axial light loss (AL2). RUO items for red blood cells (RBC), platelets (PLT) and WBC include data on hemoglobin (Hb) density, RBC/PLT sizes, distributions, immature WBCs, and so on. Several previous studies suggested that CPD parameters obtained by VCS technology can detect morphological changes in neutrophils and can provide information about the diagnosis of acute infection, sepsis, and neutrophil dysplasia [5]. These parameters capture quantitative characteristics of leukocyte morphology and activation that are not directly interpretable in isolation but may contain latent biological signals.

Unsupervised machine learning offers a systematic and powerful way to uncover hidden/subtle structures and substructures in high‐dimensional data without prior labelling. In complex clinical syndromes such as sepsis or acute respiratory distress syndrome, unsupervised clustering has revealed sub‐phenotypes with distinct outcomes and therapeutic responses, leading to new insights into disease heterogeneity and in turn to knowledge discovery from data [6].

The use of available CPD/RUO data, as well as machine learning in this context has been underexplored in the literature. A recent study utilizing CPD from the Sysmex XN‐series hematology analyzer explored their potential for the early detection of bacteremia. In this study, the fluorescent light distribution parameter of the neutrophil area (NE‐WY) was identified as a key discriminator, and machine learning approaches including decision tree modelling and support vector machines were applied to evaluate predictive performance. While these findings suggest that CPD combined with machine learning may enable earlier recognition of bacteremia, the practical integration of such approaches into routine clinical laboratory workflows remains unclear [7]. Another study concludes that integrating CPD parameters with WBC‐related suspect messages provides a sensitive, automated secondary screening strategy that reduces false negatives and enhances hematologic diagnostics (improved sensitivity/specificity to 91.9%/99.0% for blasts and 91.5%/96.7% for Immature Granulocytes (IGs), using Manual Slide Review (MSR) as the reference standard), though this integration has yet to be rolled out [8].

For this study, unsupervised machine learning clustering was applied to a large, high‐dimensional haematology dataset, including underutilised RUO and CPD, derived from a large academic hospital laboratory. The analysis was conducted on the entire all‐comer laboratory specimen database. This study aimed to develop an unsupervised global CBC/RUO/CPD phenotyping model and to evaluate, post hoc, whether thrombocytopenic specimens and clinically adjudicated thrombocytopenia mechanisms were enriched within the resulting clusters.

## 2 Method

### 2.1 Ethics

This retrospective study used de‐identified laboratory data and was approved by the institutional review board (REGIS) with waiver of consent (No. 2025/ETH00303 and No. 2025/STE00502).

### 2.2 Data Source and Cohort Definition

The study used retrospectively collected CBC data from ethylenediaminetetraacetic acid (EDTA) blood specimens analysed on Beckman Coulter DxH 900 haematology instruments (Beckman Coulter, Miami, FL, USA) between January and December 2025 at Liverpool Hospital, a single centre academic hospital laboratory. The dataset represented an all‐comer laboratory specimen cohort and was not restricted by clinical service, indication for testing, diagnostic category, or thrombocytopenia status; therefore, specimens may have originated from inpatient, outpatient, emergency, intensive care, haematology/oncology, obstetric, and paediatric settings, although these care location and specialty categories were not reliably available as model covariates and were not used for clustering. Extracted analyser files were merged into a master dataset of 537 279 rows and 183 columns; after cleaning, 537 249 laboratory results remained, and a specimen level analytical dataset was generated by retaining the first valid analyser run per specimen, yielding 254 289 unique specimens. The primary unit of analysis was therefore the specimen, not the patient. The final primary model was developed as a global all‐comer clustering model using 241 591 complete‐case specimens with 41 CBC/RUO/CPD related features; thrombocytopenia was not used to define the model, select features, fit PCA, or generate clusters. A post hoc medical record number (MRN) linkage audit successfully linked 248 346 of 254 289 specimens (97.7%) to 59 870 unique MRNs. 5943 specimens (2.3%) remained unlinked. Among linked specimens, the median, mean, and maximum numbers of specimens per MRN were 1, 4.15, and 163, respectively. The clinical annotation cohort was generated by stratified random sampling of 25 specimens from each of nine strata defined by preliminary k‐means k = 3 membership and three platelet count bands (≤ 50, > 50–≤ 100, and > 100–≤ 150 × 10^9^/L.), using random_state = 42 (*n* = 225). These specimens were subsequently mapped to the final k = 4 solution. All 225 specimen identifiers were unique, patient‐level uniqueness was not assessed.

### 2.3 Feature Selection and Missing Data

After exclusion of non‐analytic identifiers, temporal metadata, and demographic fields, 173 numeric candidate variables were available. Platelet counts were available for 254 179 specimens, with nested thrombocytopenia thresholds defined as PLT <150 × 10^9^/L (*n* = 42 503), < 100 × 10^9^/L (*n* = 20 100), and < 50 × 10^9^/L (*n* = 8889). Features with > 40% missingness were excluded, leaving 123 features for exploratory complete‐case analysis (*n* = 173 851); no imputation was performed. Three candidate feature sets were then evaluated for downstream modelling: CORE_CBC (6 features; 253 384 complete cases), CBC_EXTENDED (41 features; 241 591 complete cases), and FULL_MODEL (173 features; 0 complete cases). CBC_EXTENDED was selected as the primary feature set because it retained most specimens while preserving extended CBC/RUO/CPD information relevant to haematological phenotyping (Tables S1 and S2).

### 2.4 Unsupervised Exploration Phase

Exploratory PCA was performed on the scaled 123 feature complete‐case dataset (*n* = 173 851) to assess high‐dimensional structure before final model selection. This analysis was not used to define the final clustering model. PC1 explained 94.86% of total variance and PC2 a further 4.02% (cumulative 98.88%), producing a sharp scree‐plot elbow (Figure S1). Loading inspection showed dominance by NRBC related parameters, consistent with sparse but high contrast NRBC abnormalities disproportionately structuring the broader exploratory feature space. A 2‐D t‐distributed stochastic neighbour embedding (t‐SNE) projection of 20 000 specimens showed heterogeneous structure without clear natural separation (Figure 1). Because of this NRBC driven variance structure and complete‐case data loss, three candidate feature sets were compared: CORE_CBC (6 features), CBC_EXTENDED (41 features), and FULL_MODEL (173 features) (Table S2). CBC_EXTENDED retained 241 591 specimens (95.01%) and was selected for the final model because it preserved more of the extended CBC/RUO/CPD information while avoiding the complete‐case failure of FULL_MODEL and the limited phenotypic scope of CORE_CBC.

**FIGURE 1:**
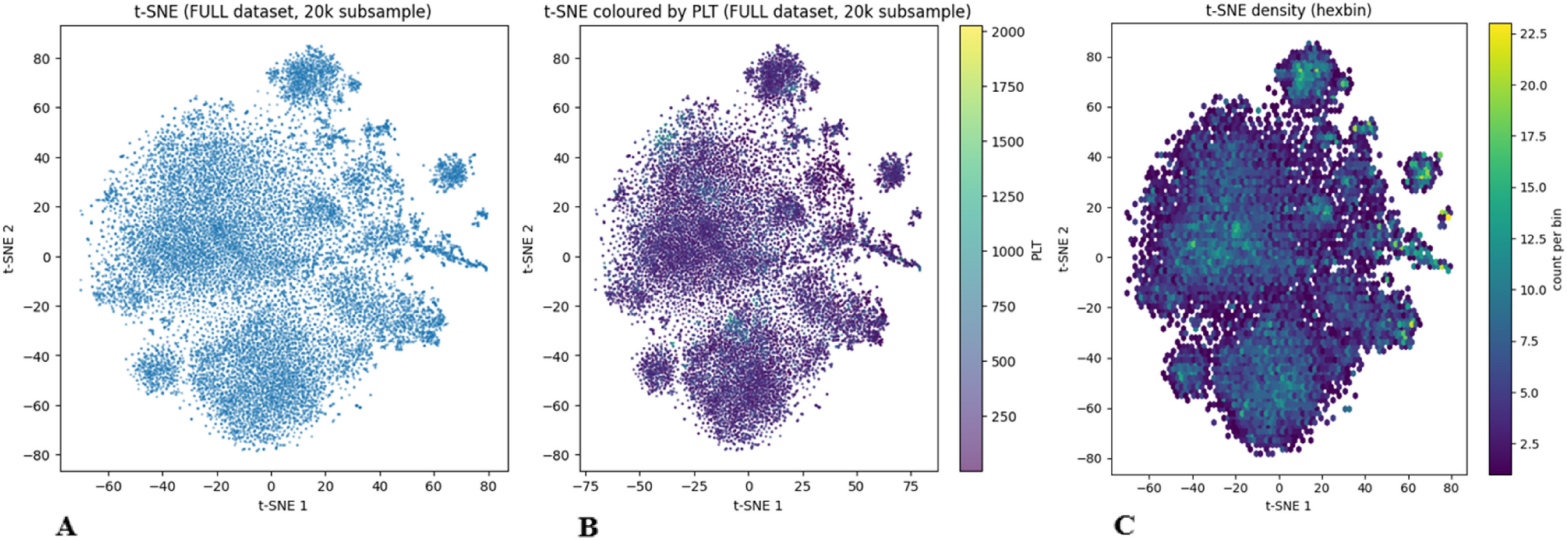
Exploratory visualization of high‐dimensional hematology data using two‐dimensional t‐distributed stochastic neighbor embedding (t‐SNE). (A) Two‐dimensional t‐SNE projection of a 20 000‐sample subset from the complete‐case dataset (123 features; *n* = 173 851) following robust scaling. Each point represents an individual specimen in the reduced nonlinear embedding space. (B) Same t‐SNE embedding coloured by platelet count (PLT), illustrating the distribution of thrombocytopenic and non‐thrombocytopenic observations across the feature space. (C) Density representation of the t‐SNE embedding using hexagonal binning, highlighting regions of high observation density within the nonlinear manifold. Together, these exploratory visualizations demonstrate heterogeneous structure in the dataset without clear natural separation, motivating refinement of the modelling feature set prior to downstream clustering.

### 2.5 Dimensionality Reduction

PCA was refitted on the CBC_EXTENDED dataset (41 features; *n* = 241 591 complete specimens) after StandardScaler normalisation to generate the final clustering feature space. The first principal component explained 20.04% of variance, and the top 20 principal components (PCA20) explained 92.94% cumulatively, indicating a substantially less single feature dominated representation than the exploratory full feature PCA. PCA loadings showed mixed contributions from platelet, red cell, NRBC related and leukocyte parameters rather than dominance by a single variable group. Bootstrap resampling [9] showed stable PCA structure, with mean Spearman loading correlations > 0.996 for PCs 1–5 across five bootstrap samples, supporting use of PCA20 for downstream clustering.

### 2.6 Clustering Methods and Internal Validation

Multiple clustering algorithms on the 20‐dimensional PCA space were evaluated, including *k*‐means [10] clustering and Gaussian mixture models (GMM) [11], across a range of cluster numbers. The study's primary clustering method was k‐means (Lloyd's algorithm with k‐means++ initialization for improved convergence) [12]. k = 2 through k = 10 clusters was explored to identify an optimal balance between over‐simplification and over‐fragmentation of phenotypes. For each *k*, internal validation metrics were computed to quantify cluster separation and compactness: (1) Silhouette coefficient (using Euclidean distance, computed on a random 10 000 sample subset for efficiency) [13], (2) Davies–Bouldin index (DB, lower values indicate better separation) [14], and (3) Calinski–Harabasz index (CH, higher values indicate better defined clusters) [15] (Table S3). The study examined the *k*‐means inertia (within‐cluster sum of squares) as an “elbow criterion” for how adding clusters improves fit [10]. These metrics were generated for *k* = 2 to 10 on a consistent subset of data to allow fair comparison across different *k* and different methods (Figure S2). Additionally, the study assessed clustering stability by bootstrap re‐sampling. For selected *k* values, the study drew multiple independent random subsets of the data (e.g., three subsamples of 30 000 specimens each), applied *k*‐means clustering to each, and then calculated the adjusted Rand index (ARI) between the cluster label assignments of each pair of subsamples. The ARI measures the agreement between two partitions, adjusted for chance (ARI = 1 indicates identical clustering, ARI ~ 0 expected for random agreement) [16]. A high mean ARI across bootstrap runs indicates that the clustering solution is reproducible and not sensitive to sampling variability.

In parallel, the study applied Gaussian mixture models (GMM) with full covariance matrices as an alternative approach for *k* = 2 to 10, to see if elliptical Gaussian clusters would better fit the data distribution. GMM clustering was similarly evaluated with the previously mentioned internal metrics and stability checks (Table S3). The study also briefly explored density based clustering with DBSCAN [17] and graph based clustering with spectral clustering for comparison [18]. For DBSCAN, the *k*‐distance graph method was used to choose an epsilon parameter: distances to the 15 th nearest neighbour were plotted in sorted order for a sample of the data to identify a “knee” in the distance curve as a heuristic for ε. The study examined quantiles of this distance distribution (e.g., 90th percentile ~ 92.8, 95th ~105.0, 97.5th ~118.0, 99th ~135.9 in the distance plot) and a range of ε values were tried around these; the minimum points (min_samples) were set to 15 (Figure S3). Spectral clustering (unnormalized Laplacian, with *k* = 4 and 10 nearest neighbours for affinity) was attempted on a smaller subsample (*n* ≈ 12 000, due to computational limits of spectral decomposition). We did not pursue DBSCAN or spectral clustering for the full analysis, due to result and computational limitations. Instead, we focused on *k*‐means (primary method) and GMM (secondary method) as our clustering approaches (Table 1).

**TABLE 1:** Candidate clustering model comparison on a fixed subsample. Internal clustering performance metrics are shown for candidate k‐means and Gaussian mixture model (GMM) solutions evaluated in PCA20 feature space using a fixed random subsample of 60 000 complete‐case specimens. For this model‐comparison table, the silhouette coefficient, Davies–Bouldin index and Calinski–Harabasz index were all computed within the same 60 000‐specimen subsample. The k‐means model with k = 4 demonstrated comparatively favourable internal metrics relative to the evaluated GMM models and was retained as the primary clustering approach for downstream analyses.

Model	*n*	Silhouette	Davies_Bouldin	Calinski_Harabasz
KMeans_k4	60 000	0.10	2.21	6536.92
GMM_k3	60 000	0.08	4.69	2072.38
GMM_k4	60 000	0.09	3.98	2547.86

### 2.7 Selection of Optimal Clustering

The optimal number of clusters was determined using internal validation and stability analyses (Table S3, Table 1). K‐means solutions were evaluated across multiple k values using silhouette coefficient, Davies–Bouldin index, Calinski–Harabasz index, inertia trends, and bootstrap reproducibility assessed via adjusted Rand index (ARI). Although silhouette values decreased gradually with increasing k and no single elbow was observed in inertia plots (Figure S2). Internal validation indices were mixed and did not identify a single unequivocal optimum. The k = 4 solution was selected based on a composite assessment of internal validation, stability, parsimony, and interpretability. Post hoc clinical annotation was performed only after the global model had been generated. Solutions with k ≥ 5 demonstrated reduced reproducibility and increased fragmentation into small clusters.

The final k‐means model (k = 4) was fitted on the PCA20 feature space (*n* = 241 591) using k‐means++ initialization with 50 random starts (“n_init = 50”). Cluster assignments were merged with pre‐scaled laboratory data for plausibility assessment. Cluster‐level summary statistics were calculated for selected CBC parameters as medians and interquartile ranges. Overall, between cluster differences were evaluated using Kruskal‐Wallis tests, and epsilon‐squared (ε^2^) was calculated as a non‐parametric effect size measure to aid interpretation in the context of the large sample size (Table S4). To assess potential technical confounding, cluster membership was examined across analyzer platforms and time. These validation procedures were undertaken to ensure that clustering structure reflected biological signal rather than batch or instrumentation effects.

### 2.8 Cluster Characterization and Statistical Analysis

Cluster phenotypes were profiled using cluster sizes and median laboratory values, with global CBC parameter distributions reported in Table S4. Thrombocytopenia was evaluated post hoc by applying nested platelet thresholds (PLT < 150, < 100 and < 50 × 10^9^/L) to the generated k = 4 cluster assignments and examining threshold‐specific cluster composition and laboratory profiles (Figures S7 and S8). Exploratory thrombocytopenia restricted k‐means analyses were also performed within platelet defined subsets as sensitivity analyses, but were not used to define the primary model (Table S1, Figure S4). Statistical comparisons within each subset were performed using Kruskal–Wallis tests [19] (to assess overall cluster differences in, for example, WBC or MPV within just the PLT < 50 cohort) followed by post hoc pairwise Mann–Whitney U‐tests with Benjamini–Hochberg [20] false discovery rate correction. Finally, the rank‐biserial effect sizes [21] for each pairwise comparison to quantify the magnitude of differences was calculated. Thrombocytopenic specimens were not used to train a separate primary clustering model, thrombocytopenia severity and EHR identified primary mechanism categories were evaluated only after mapping specimens back onto the global k = 4 solution.

### 2.9 Post Hoc Clinical Annotation and Evaluation

Post hoc clinical annotation was performed using 225 thrombocytopenic specimens with expert adjudicated “EHR_Mechanism_Primary” labels derived from EHR progress note review. Cases were categorised as “Underproductive,” “Overdestructive,” “Other,” or “Mixed,” and their cluster‐wise CBC profiles are summarised in Table S5. Cases were mapped onto the generated k = 4 cluster solution without model retraining. Concordance between cluster assignments and adjudicated mechanisms was assessed using purity, adjusted Rand index (ARI) [16], normalized mutual information (NMI) [22], and the Variation of Information (VI) [23], adjusted mutual information, χ^2^ testing, and Cramér's V. Sensitivity analyses assessed label heterogeneity by excluding Mixed cases (*n* = 204), excluding Other cases (*n* = 188), and restricting analysis to Underproductive versus Overdestructive cases (*n* = 167) (Table 2; Tables S6–S8).

**TABLE 2:** Post hoc clinical annotation/evaluation sensitivity analyses across clinical label subsets. Metrics for clustering models evaluated against clinically adjudicated thrombocytopenia mechanisms in the post hoc clinical annotation/evaluation. Agreement between model‐derived clusters and EHR‐derived mechanism labels was assessed using cluster purity, adjusted Rand index (ARI), and normalized mutual information (NMI). Analyses were performed on the full cohort (ALL, *n* = 225) and across progressively restricted subsets to assess the influence of heterogeneous or uncertain clinical labels: EXCLUDE_OTHER (removing cases classified as “Other”), EXCLUDE_MIXED (removing mixed‐mechanism cases), and CORE_ONLY (retaining only core mechanisms of overdestruction and underproduction). Across all scenarios, the k‐means k = 4 model demonstrated the highest or comparable concordance with clinical labels relative to Gaussian mixture models (GMM), with purity increasing as heterogeneous mechanisms were excluded. These findings support the robustness of the k = 4 clustering solution across clinically defined sensitivity analyses.

Model	Analysis	*n*	Purity	ARI	NMI
KMeans_k4	ALL	225	0.59	0.14	0.18
KMeans_k4	EXCLUDE_OTHER	188	0.63	0.12	0.11
KMeans_k4	EXCLUDE_MIXED	204	0.65	0.16	0.20
KMeans_k4	CORE_ONLY	167	0.71	0.13	0.11
GMM_k3	ALL	225	0.52	0.08	0.08
GMM_k3	EXCLUDE_OTHER	188	0.62	0.08	0.09
GMM_k3	EXCLUDE_MIXED	204	0.57	0.10	0.09
GMM_k3	CORE_ONLY	167	0.69	0.11	0.10
GMM_k4	ALL	225	0.49	0.05	0.09
GMM_k4	EXCLUDE_OTHER	188	0.59	0.05	0.06
GMM_k4	EXCLUDE_MIXED	204	0.54	0.07	0.09
GMM_k4	CORE_ONLY	167	0.66	0.07	0.07

### 2.10 Statistical Software

All analyses were conducted in Python using Pandas for data processing, scikit‐learn for scaling, PCA, k‐means and GMM clustering, and SciPy for statistical testing. Visualizations were generated using Matplotlib and Seaborn. The study followed TRIPOD‐AI‐informed reporting principles where applicable to transparent model development and evaluation. Clinical annotation cases were reserved for post hoc clinical annotation/evaluation and were not used for model fitting.

## 3 Results

### 3.1 Exploration Phase

These analyses were exploratory and were not used to define the final clustering model. After data integration and cleaning, the specimen level dataset contained 254 289 unique results with 173 numeric candidate variables. Removal of 50 features with > 40% missingness left 123 features, yielding 173 851 complete‐case specimens for exploratory PCA. PC1 explained 94.9% of total variance and PC2 a further 4.0%, indicating a dominant variance axis driven largely by NRBC related measures (Figure S1). Exploratory clustering in this broader PCA space showed limited natural partitioning, substantial overlap beyond PC1, and no clear clustering optimum (Figures S2 and S5). Exploratory k‐means analyses, including thrombocytopenia restricted sensitivity analyses, identified leukocyte and erythrocyte associated patterns and small outlier groups, including severe leukocytosis and pancytopenic profiles (Figures S4 and S6, Table S1).

### 3.2 Development and Evaluation Phase

The final global CBC_EXTENDED model included 241 591 complete‐case specimens and generated four broad haematological clusters. The CBC_EXTENDED feature set (41 features) was selected because it retained 95.0% of specimens while preserving extended CBC/RUO/CPD information; by comparison, CORE_CBC retained 99.6% of specimens but excluded morphological parameters, whereas the 173 feature FULL_MODEL yielded no complete cases (Table S2). Complete‐case bias analysis showed statistically significant but small differences between retained and excluded cases, supporting use of CBC_EXTENDED for downstream modelling (Table S2). PCA20 of the refined feature space was used for clustering, and internal validation supported k = 4 as a more stable and interpretable solution, with favourable Calinski–Harabasz and Davies–Bouldin indices relative to adjacent k values (Table 1; Table S3). The final k = 4 model produced four clusters comprising 14%–37% of specimens, with significant differences across core CBC parameters and distinct global CBC/RUO/CPD profiles (Table S4; Figure 2). Assessment of instrument and extraction batch variables showed no evidence that complete‐case retention or cluster membership was driven by analyser or extraction batch effects.

**FIGURE 2:**
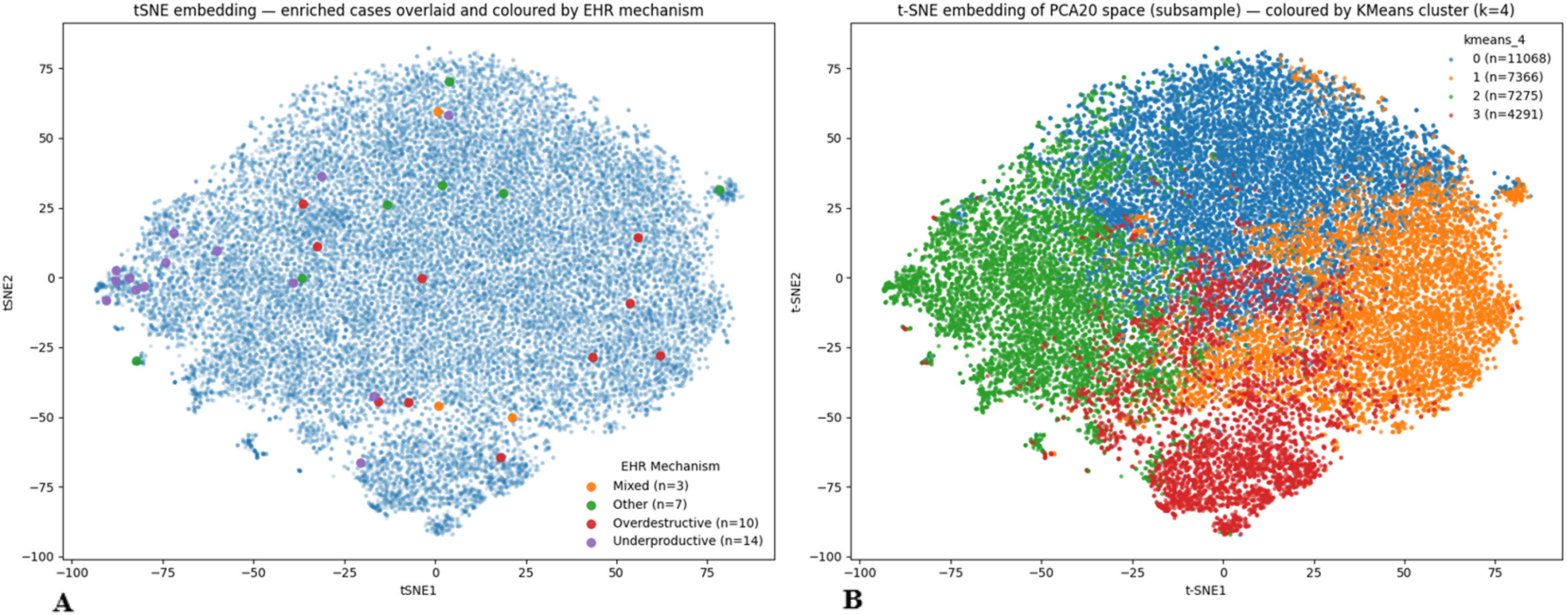
Global phenotypic structure of clusters and clinical enrichment in t‐SNE space. Two‐dimensional t‐distributed stochastic neighbour embedding (t‐SNE) of the PCA20 feature space is shown for a subsample of specimens (*n* = 30 000) derived from the global dataset (*n* = 241 591). (A) Clinically annotated thrombocytopenic cases present within the t‐SNE subsample (*n* = 34) are overlaid on the background distribution and coloured by clinically adjudicated thrombocytopenia mechanism from electronic health record review (Mixed *n* = 3, Other *n* = 7, Overdestructive *n* = 10, Underproductive *n* = 14). Background points represent the remaining subsampled specimens shown in light grey. (B) The same t‐SNE embedding coloured by cluster assignment from the final k‐means model (k = 4), illustrating the spatial organization of unsupervised phenotypic clusters within the reduced‐dimensional feature space (cluster sizes in the subsample: C0 = 11 068; C1 = 7366; C2 = 7275; C3 = 4291). Together, these projections illustrate the global structure of hematologic phenotypes and the distribution of clinically adjudicated cases within the unsupervised cluster landscape.

Thrombocytopenia was then evaluated post hoc within the final global k = 4 cluster map. Across nested platelet thresholds (PLT < 150, < 100, and < 50 × 10^9^/L), Cluster 3 showed progressive enrichment, accounting for approximately 46%, 60%, and 71% of threshold defined cases, respectively, while all four clusters remained represented (Figures S7 and S8). Separate thrombocytopenia restricted exploratory k‐means sensitivity analyses (k = 5), generated within each platelet‐defined subset using the CBC_EXTENDED complete‐case feature space, also demonstrated persistent phenotypic structure, including small severe leukocytosis outlier clusters and broader pancytopenic profiles; these analyses were not used to select, train, or interpret the final global k = 4 model (Table S1). Figure S4 visualises the broader 123 feature exploratory analysis.

Post hoc clinical annotation was performed in 225 unique thrombocytopenic specimens, all of which mapped to the final global k = 4 assignments. Concordance between unsupervised clusters and adjudicated EHR_Mechanism_Primary labels was limited but showed partial and non‐random enrichment: K‐means k = 4 achieved purity 0.587, ARI 0.144, and NMI 0.180, (adjusted for chance) with sensitivity analyses showing modest improvement after excluding Mixed cases or restricting to selected mechanism categories (Table 2; Tables S6–S8). These findings indicate partial alignment between data driven CBC/RUO/CPD phenotypes and adjudicated thrombocytopenia mechanisms, rather than direct classification of clinical aetiology (Figure 3).

**FIGURE 3:**
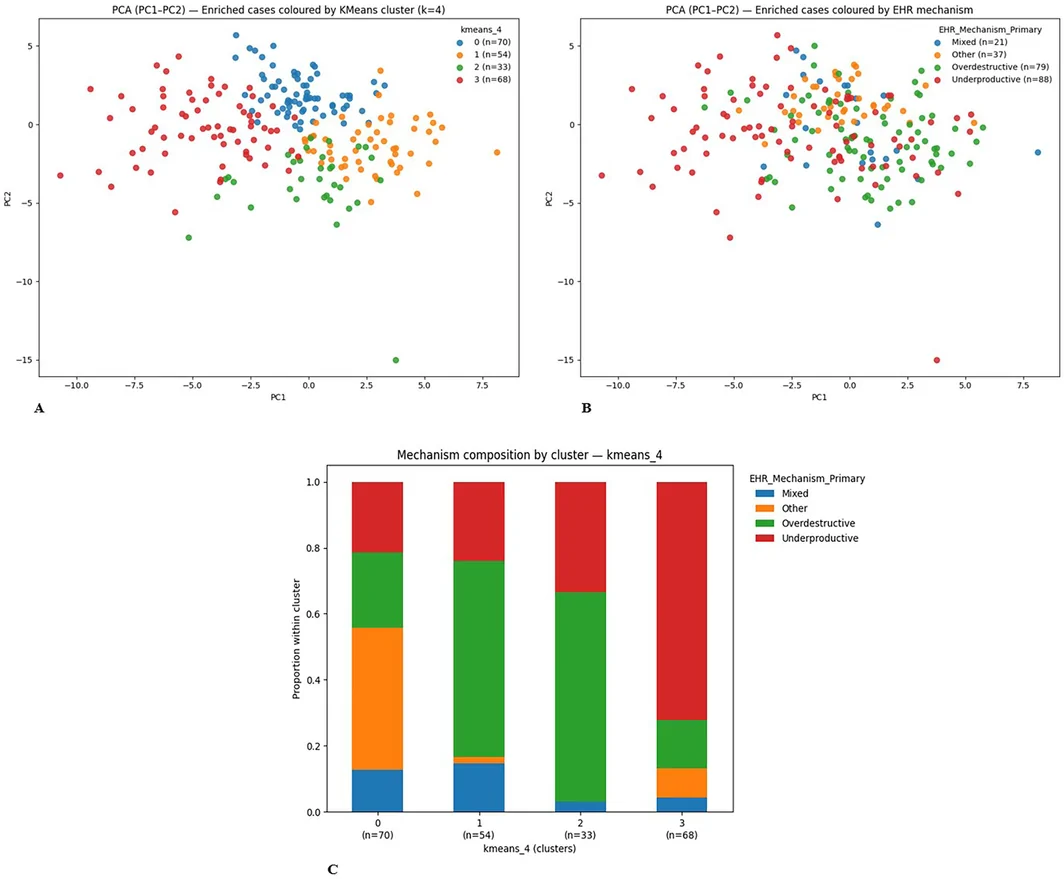
Relationship between unsupervised clusters and clinically adjudicated thrombocytopenia mechanisms in the post hoc clinical annotation cohort. Clinically annotated cases (*n* = 225) were projected into the first two principal components of the global PCA model derived from the full dataset (*n* = 241 591 specimens). (A) Scatter plot of enriched cases coloured by cluster assignment from the final k‐means model (k = 4) (C0 = 70, C1 = 54, C2 = 33, C3 = 68). (B) The same PCA projection coloured by clinically adjudicated thrombocytopenia mechanism obtained from electronic health record review (Underproductive *n* = 88, Overdestructive *n* = 79, Other *n* = 37, Mixed *n* = 21). (C) Stacked bar plot showing the distribution of clinical mechanisms within each cluster. Clusters demonstrated differing mechanism enrichment, with Cluster 3 predominantly underproductive thrombocytopenia (49/68, 72%), clusters 1 and 2 enriched for overdestructive mechanisms (32/54, 59% and 21/33, 64%, respectively), and cluster 0 demonstrating greater mechanistic heterogeneity. Together, these analyses demonstrate partial concordance between unsupervised hematologic phenotypes and clinically adjudicated thrombocytopenia mechanisms.

## 4 Discussion

This study demonstrates that unsupervised clustering of all‐comer CBC/RUO/CPD data can identify broad haematological phenotypes and shows reproducible structure within which thrombocytopenia severity and adjudicated thrombocytopenia mechanisms show partial enrichment. The final model was developed from 241 591 complete‐case specimens using the CBC_EXTENDED feature set and was not trained on thrombocytopenia labels or clinical mechanisms. Thus, the main finding is not that the model directly classified thrombocytopenia aetiology, but that high‐dimensional routine analyser data contained reproducible laboratory structure that could be evaluated post hoc against platelet thresholds and clinically adjudicated mechanisms.

The four global clusters represented broad CBC/RUO/CPD phenotypes rather than discrete diagnostic categories. Cluster 3 resembled a cytopenic/lower platelet profile in the global model and became progressively enriched across increasing thrombocytopenia severity, accounting for approximately 46%, 60%, and 71% of specimens with PLT < 150, < 100, and < 50 × 10^9^/L, respectively (Figures S7 and S8). In the post hoc clinical annotation cohort, Cluster 3 was also enriched for underproductive mechanisms, whereas overdestructive mechanisms were distributed mainly across Clusters 1 and 2, and Cluster 0 remained more heterogeneous (Figure 3; Tables S5–S8). These findings support partial enrichment of clinically interpretable patterns, but not direct recovery of clinical aetiology classes.

The utility of this approach lies in the ability to use underutilised information already produced by modern haematology analysers. Machine learning algorithms can integrate subtle multivariable patterns across CBC, RUO, and CPD parameters that may not be apparent when individual indices are considered in isolation. This is important because these data are generated as part of routine CBC analysis and do not require additional sample collection, laboratory testing, or patient burden. In this sense, the current study provides a first step framework for laboratory phenotyping and hypothesis generation using routinely available high‐dimensional analyser outputs.

Our data driven phenotypes resonate with findings in other fields, where unsupervised clustering has identified clinically relevant subgroups in heterogeneous syndromes [5, 7, 8, 24, 25, 26, 27, 28, 29, 30, 31]. In critical care, unsupervised clustering has identified heart failure and thrombocytopenia subphenotypes with differing outcomes or treatment associations [26, 32]. However, those studies often incorporated broad clinical variables or focused on specific clinical settings [6, 26]. In contrast, the present study shows that haematology analyser data alone, derived from a single routine laboratory assay, contains sufficient structure to support exploratory phenotyping in a large all‐comer hospital specimen cohort.

The findings also reinforce the limitation of relying on single platelet indices to infer thrombocytopenia mechanisms. MPV has long been investigated as a potential discriminator between immune and hypoproductive thrombocytopenia [33, 34, 35], and meta‐analytic data suggest higher MPV in ITP than hypoproductive thrombocytopenia, although with only moderate diagnostic accuracy [35]. In the present study, MPV differed only modestly across the global k = 4 clusters, with median MPV ranging from 8.26 to 8.51 fL, and the Cluster 2 versus Cluster 3 difference in the post hoc clinical annotation cohort was 0.63 fL. Therefore, MPV alone does not explain the observed structure; rather, the clusters reflect multivariable CBC/RUO/CPD patterns. Similarly, PCT and other extended parameters may contribute useful contextual information, but their value is likely greatest when interpreted as part of an integrated high‐dimensional pattern rather than as standalone diagnostic markers [34, 35, 36, 37].

The modest internal separation of the final k = 4 solution is important for interpretation. The sampled silhouette score was approximately 0.10, indicating substantial overlap between clusters. Davies–Bouldin and Calinski–Harabasz indices supported comparison across candidate solutions, but they do not establish strong biological separation. Accordingly, the clusters should be interpreted as aggregate laboratory phenotypes rather than sharply separated disease classes. The exploratory DBSCAN analysis also did not identify clearly separated density‐based clusters, supporting the interpretation that thrombocytopenia‐associated laboratory patterns exist on a continuum rather than as discrete silos.

Post hoc clinical annotation showed partial alignment between unsupervised clusters and adjudicated labels (adjusted for random alignment). K‐means k = 4 achieved purity 0.59, ARI 0.14, and NMI 0.18 in the full 225‐case annotation cohort, with modest improvement in selected sensitivity analyses after excluding mixed or heterogeneous labels (Table 2; Tables S6–S8). These values indicate partial enrichment rather than diagnostic classification. This level of agreement is consistent with the expected difficulty of mapping unsupervised laboratory phenotypes onto retrospective clinical mechanism labels, particularly when cases may have mixed mechanisms, uncertain documentation, or overlapping haematological features [38, 39].

Several limitations should therefore be noted. This was a retrospective, single centre, specimen level study using one analyser platform model, and the clusters may reflect local case mix, instrument specific characteristics/measurement sensitivities, and repeated testing in clinically complex patients. Although MRN linkage showed that many linked patients contributed a single specimen, some patients contributed multiple specimens, and patient level sensitivity analyses were not performed. The model was not adjusted for age, sex/gender, or ethnicity; age and sex/gender were available in linked metadata but were not included because the clustering model was restricted to analyser derived CBC/RUO/CPD variables, and ethnicity was not reliably available. The post hoc clinical annotation cohort was relatively small, retrospective, and based on progress note review, so label uncertainty and mixed mechanisms likely limited ARI and NMI. Some rare thrombocytopenia aetiologies were under represented and may not have formed distinct phenotypic groups.

Despite these limitations, the study provides a reproducible proof‐of‐concept framework for unsupervised phenotyping from high‐dimensional routine haematology data. The results support the premise that underutilised CBC/RUO/CPD parameters contain clinically relevant phenotypic signal, even when no diagnostic labels are used for model development. Future work should evaluate this framework in prospective, patient level, multi‐centre cohorts, incorporate demographic and longitudinal information, and determine whether these laboratory phenotypes improve hypothesis generation, diagnostic prioritisation, or downstream clinical research when combined with clinical context.

## 5 Conclusion

This study demonstrates that unsupervised clustering of all‐comer CBC/RUO/CPD data can identify broad, reproducible haematological phenotypes using information already generated during routine haematology analysis. The final global CBC_EXTENDED k = 4 model identified laboratory phenotypes with post hoc enrichment for thrombocytopenia severity and adjudicated EHR_Mechanism_Primary categories. These findings support the utility of underutilised analyser derived parameters for laboratory phenotyping and hypothesis generation. However, cluster membership should not be interpreted as a standalone diagnostic assignment or used to guide clinical decisions without further validation. Future prospective, patient‐level, multi‐centre studies are required to determine whether these phenotypes can improve diagnostic prioritisation, laboratory workflows, or clinical and laboratory diagnostic research in thrombocytopenia and broader haematology.

## Author Contributions

M.A.A. conceived and designed the study, performed data processing and machine learning model development, and drafted the manuscript. E.J.F. and A.B.S. provided domain expertise in hematology and hemostasis, contributed to study design and feasibility considerations, and critically structured and revised the manuscript. M.Z.I. provided methodological guidance in machine learning framework development and assisted with model evaluation and validation. S.N. and D.H. Consultant Haematologists, critically reviewed the manuscript, including evaluation of the clinical implications of the findings and contributions from a haematology clinical perspective. All authors critically reviewed multiple drafts of the manuscript, contributed to interpretation of the findings, and approved the final version.

## Funding

The authors have nothing to report.

## Conflicts of Interest

The authors declare no conflicts of interest.

## Supporting information


**Figure S1:** PCA cumulative explained variance scree plot. Cumulative proportion of variance explained by successive principal components derived from the scaled complete‐case dataset (123 features; *n* = 173 851). The first principal component accounts for the majority of variance, producing a sharp elbow in the scree curve and indicating that a small number of components capture most of the variability in the full feature space.
**Figure S2:** Internal diagnostic plots for selection of cluster number in k‐means clustering. (A) Elbow plot showing k‐means inertia (within‐cluster sum of squares) across candidate cluster numbers (k = 2–15) in the 20‐dimensional PCA feature space, illustrating the diminishing improvement in model fit with increasing k. (B) Silhouette coefficient as a function of cluster number (k), computed using Euclidean distance on a random 10 000‐sample subset, showing progressive decline in average cluster separation as k increases. These diagnostic plots were used alongside other internal validation metrics to assess clustering structure and guide selection of an appropriate cluster number.
**Figure S3:** k‐nearest neighbour distance plot for DBSCAN parameter estimation. Sorted k‐NN distance plot (k = 15) computed in the 20‐dimensional PCA feature space using a subsample of 50 000 observations. Distances to the 15th nearest neighbour are plotted in ascending order to identify a potential “knee” in the curve for selection of the DBSCAN ε parameter. Quantile thresholds of the distance distribution are shown for reference (90th ≈ 92.8, 95th ≈ 105.0, 97.5th ≈ 118.0, 99th ≈ 135.9), which were explored as candidate ε values during density‐based clustering experiments.
**Figure S4:** Sensitivity analysis of thrombocytopenia subsets using k‐means clustering (k = 5). Visualization of exploratory clustering using the broader 123‐feature analysis performed separately within thrombocytopenia cohorts defined by platelet count thresholds (< 150, < 100, and < 50 × 10^9^/L). For each cohort, numerical CBC and CPD features were standardized and reduced using principal component analysis (PCA), retaining the first 20 principal components (PCA20) prior to clustering. K‐means clustering (k = 5, k‐means++ initialization, n_init = 20, random_state = 42) was applied within each subset to assess whether additional phenotypic structure emerged under varying thrombocytopenia severity thresholds. (A) PCA projection (PC1 vs. PC2) of the PLT < 150 × 10^9^/L subset colored by k‐means cluster assignments. (B) Two‐dimensional t‐SNE embedding of the same cohort (subsample visualization) colored by cluster labels. (C) PCA projection (PC1 vs. PC2) for the PLT < 100 × 10^9^/L subset following PCA20 dimensionality reduction and k‐means clustering. (D) Corresponding t‐SNE visualization of the PLT < 100 cohort colored by cluster membership. (E) PCA projection (PC1 vs. PC2) for the PLT < 50 × 10^9^/L subset representing severe thrombocytopenia. (F) Corresponding t‐SNE embedding showing cluster structure within this severe cohort. Across all severity strata, clusters demonstrated partially separable structure in PCA space with broadly consistent organization in t‐SNE embeddings, although cluster dispersion increased as platelet thresholds decreased due to reduced sample size. These analyses were performed as sensitivity checks to evaluate clustering stability across thrombocytopenia severity definitions and were not used for the final phenotypic model reported in the main analysis.
**Figure S5:** Exploratory visualization of k‐means clustering in the full complete‐case PCA feature space. K‐means clustering (k = 5) was applied as an exploratory analysis to the full complete‐case dataset represented in the 20‐dimensional PCA space (*n* = 173 851 specimens). (A) Projection of clusters onto PC1 versus PC2. (B) Overlay of the same cluster assignments onto a t‐SNE embedding generated from a 20 000‐specimen subsample. (C) Alternate rendering of the PC1 versus PC2 projection with cluster colour scale. (D) Projection of clusters onto PC1 versus PC3. (E) Projection of clusters onto PC2 versus PC3. Across pairwise PCA views, separation was dominated primarily along PC1, whereas substantial overlap persisted in secondary component space and in the t‐SNE embedding. These exploratory visualizations support the interpretation that, in the full feature set, clustering structure was driven largely by a dominant variance axis rather than by clearly separated natural groups.
**Figure S6:** Exploratory k‐means clustering (k = 5) in the complete‐case PCA space and t‐SNE projection.
(A) K‐means clustering with k = 5 applied to the complete‐case PCA representation of the broader exploratory feature set (*n* = 173 851), shown in PC1–PC2 space. (B) The same exploratory clustering visualized on a t‐SNE embedding derived from a 20 000‐case subsample. These analyses were used for exploratory structure assessment and were not the final clustering model used for post hoc clinical annotation/evaluation.
**Figure S7:** Sensitivity analysis of the final global k‐means clustering solution across thrombocytopenia severity thresholds. Boxplots show distributions of platelet count (PLT), white blood cell count (WBC; log10[WBC + 0.01]), mean platelet volume (MPV), and plateletcrit (@PCT) across clusters from the final global CBC_EXTENDED k‐means model (k = 4) after restriction to progressively thrombocytopenic complete‐case subsets. The PLT < 100 × 10^9^/L subset included 18 077 specimens, with cluster sizes C0 = 3120, C1 = 2569, C2 = 1508, and C3 = 10 880. The PLT < 50 × 10^9^/L subset included 7562 specimens, with cluster sizes C0 = 966, C1 = 748, C2 = 487, and C3 = 5361. Boxes represent interquartile ranges with medians indicated. Cluster 3 was progressively enriched across thrombocytopenia thresholds, supporting its interpretation as the global cytopenic/lower‐platelet phenotype within the final k = 4 model.
**Figure S8:** Distribution of final global k‐means clusters across thrombocytopenia severity thresholds. Stacked bar plots show the proportion of complete‐case specimens assigned to each cluster from the final global CBC_EXTENDED k‐means model (k = 4) across nested platelet count thresholds (PLT < 150, < 100, and < 50 × 10^9^/L). Sample sizes were *n* = 39 333, *n* = 18 077, and *n* = 7562, respectively. Cluster 3 accounted for 18 147/39333 (46.14%) PLT < 150 cases, 10 880/18077 (60.19%) PLT < 100 cases, and 5361/7562 (70.89%) PLT < 50 cases, demonstrating progressive enrichment of Cluster 3 with increasing thrombocytopenia severity. These findings show that thrombocytopenic specimens are preferentially represented in Cluster 3 within the final global CBC_EXTENDED k = 4 clustering solution.
**Table S1:** Generated thrombocytopenia restricted exploratory k‐means sensitivity analyses across two feature spaces. K‐means clustering (k = 5) was performed separately within nested platelet defined thrombocytopenia subsets (PLT < 150, < 100, and < 50 × 10^9^/L) using two independently fitted feature spaces: the CBC_EXTENDED 41‐feature complete‐case feature set used in the primary global modelling workflow, and a broader missingness‐filtered 123‐feature exploratory complete‐case feature set used for supplementary sensitivity analysis. Within each platelet defined subset and feature space, variables were scaled, reduced using principal component analysis with 20 components, and clustered using k‐means++ initialisation with n_init = 20 and random_state = 42. CBC_EXTENDED complete‐case subset sizes were 39 333 for PLT < 150 × 10^9^/L, 18077 for PLT < 100 × 10^9^/L, and 7562 for PLT < 50 × 10^9^/L. The corresponding broader exploratory complete‐case subset sizes were 29 625, 13 732, and 5412, respectively. Values are shown as cluster size, platelet median [25th–75th percentile], WBC median, and RBC median. Because clustering was fitted independently within each feature space and platelet threshold, cluster numbers are model specific and should not be interpreted as matched across feature spaces, platelet thresholds, or the final global k = 4 model. These analyses were exploratory sensitivity assessments and were not used to select, train, validate, or interpret the final global k = 4 CBC_EXTENDED model.
**Table S2:** Candidate feature‐set definitions, complete‐case retention and complete‐case bias analysis. Complete‐case retention was evaluated across three candidate feature sets constructed from 254 289 specimen‐level records: CORE_CBC, comprising six standard CBC parameters; CBC_EXTENDED, comprising 41 CBC/RUO/CPD‐related parameters used for the primary global clustering model; and FULL_MODEL, comprising 173 numeric parameters after exclusion of non‐analytic identifiers and date/time fields. CORE_CBC retained 253 384 specimens (99.64%), CBC_EXTENDED retained 241 591 specimens (95.01%), and FULL_MODEL yielded no complete cases because of pervasive missingness across research parameters. To assess potential selection bias from complete‐case filtering, retained and excluded specimens were compared for PLT, WBC, RBC, HGB, MCV and RDW using medians and Mann–Whitney U tests. Although statistically significant differences were observed, reflecting the large sample size, absolute differences for the CBC_EXTENDED feature set were small and not suggestive of clinically meaningful distortion. For FULL_MODEL, retained‐case medians and Mann–Whitney U *p*‐values were not applicable.
**Table S3:** Internal validation metrics for k‐means cluster‐number assessment in the full PCA20 cohort. K‐means models were fitted across k = 2–10 clusters in the 20‐dimensional PCA feature space of the full CBC_EXTENDED complete‐case cohort (*n* = 241 591). The silhouette coefficient was computed on a fixed random subset of 10 000 specimens for computational feasibility, whereas the Davies–Bouldin index, Calinski–Harabasz index and model inertia were computed on the full PCA20 cohort. These metrics were used as internal diagnostics to compare candidate k values and guide selection of an interpretable clustering solution.
**Table S4:** Cluster‐wise distributions of CBC parameters across the final global k‐means model (k = 4). Median values and interquartile ranges (IQRs) are shown for 23 CBC parameters across the four clusters identified by the final global CBC_EXTENDED k‐means model. The model was fitted in PCA20 feature space using 241 591 complete‐case specimens. Cluster sizes were 89 364 for cluster 0, 58 618 for cluster 1, 34 858 for cluster 2 and 58 751 for cluster 3. Overall, between‐cluster differences were assessed using Kruskal–Wallis tests based on available non‐missing values for each parameter, with epsilon‐squared (ε^2^) reported as a non‐parametric effect‐size measure. These summaries were used to define the global cluster phenotypes before assessing post hoc thrombocytopenia and clinical‐mechanism enrichment.
**Table S5:** Cluster‐wise haematological profiles in the post hoc clinical annotation cohort. Values are median [interquartile range] for selected complete blood count and platelet parameters across the four clusters in the 225‐specimen post hoc clinical annotation cohort mapped to the final global k = 4 solution. PLT, platelet count; WCC, white cell count; MPV, mean platelet volume; @PCT, plateletcrit. Overall, between‐cluster differences were assessed using Kruskal‐Wallis tests. Raw and Benjamini‐Hochberg (BH)‐adjusted *p*‐values are reported, with BH adjustment applied across the four tested features; epsilon‐squared (ε^2^) is the omnibus effect‐size estimate. Patient‐level independence was not established. No pairwise comparisons were undertaken. Cluster assignments should be interpreted as post hoc phenotypic annotations.
**Table S6:** Post hoc clinical annotation/evaluation metrics for unsupervised clustering models. Performance metrics comparing unsupervised clustering assignments with clinically adjudicated thrombocytopenia mechanisms in the post hoc clinical annotation/evaluation cohort (*n* = 225). Agreement between model‐derived clusters and EHR‐derived mechanism labels was assessed using cluster purity, adjusted Rand index (ARI), and normalized mutual information (NMI). Among models compared post hoc against adjudicated mechanism labels, k‐means k = 4 showed the highest concordance, although agreement remained limited. These findings indicate partial clinical enrichment of the selected global k = 4 solution rather than external validation or diagnostic classification.
**Table S7:** Comparative summary of clustering model performance against clinically adjudicated thrombocytopenia mechanisms. Comparison of unsupervised clustering approaches evaluated against EHR‐derived thrombocytopenia mechanism labels in the post hoc clinical annotation/evaluation cohort. Agreement between model‐derived clusters and clinical labels was assessed using cluster purity, adjusted Rand index (ARI), and normalized mutual information (NMI). Gaussian mixture models (GMM; k = 3 and k = 4) and k‐means (k = 4) were fitted in the PCA20 feature space of the full dataset and evaluated on the 225 cases. An exploratory spectral clustering model (k = 4) was applied to a 12 000‐specimen subsample, resulting in overlap with only 13 enriched cases, and is therefore presented for qualitative comparison only. Across models evaluated on the full post hoc clinical annotation cohort, k‐means (k = 4) demonstrated the strongest concordance with clinical labels (purity = 0.59, ARI = 0.15, NMI = 0.18). Rank columns indicate relative model performance across the three evaluation metrics.
**Table S8:** Concordance and statistical association between clustering models and clinically adjudicated thrombocytopenia mechanisms. Agreement between unsupervised clustering assignments and clinically adjudicated thrombocytopenia mechanisms in the post hoc clinical annotation/evaluation cohort (*n* = 225) was evaluated using multiple complementary metrics, including the adjusted Rand index (ARI), adjusted mutual information (AMI), and V‐measure. Statistical association between cluster membership and mechanism labels was additionally assessed using the chi‐square test of independence and Cramér's V as a measure of effect size. Across models evaluated on the full PCA20 feature space, the k‐means k = 4 model demonstrated the strongest concordance with clinical labels, with moderate association strength (Cramér's V = 0.397) and highly significant association (χ^2^ *p* = 9.0 × 10^−19^), whereas Gaussian mixture models showed weaker concordance. Noise fraction represents the proportion of enriched cases assigned to clusters inconsistent with their clinically adjudicated mechanism.

## Data Availability

The data that support the findings of this study are not publicly available due to ethical and privacy restrictions. The dataset contains de‐identified patient laboratory data obtained under institutional ethics approval, and release of these data is not permitted under the terms of that approval or applicable privacy regulations.
