# Supplementary material for: Unsupervised Global CBC/RUO/CPD Phenotyping Identifies Haematological Clusters Enriched for Thrombocytopenia Severity and Mechanisms
Source: Int J Lab Hematol. 2026 Aug 11;48(6):1385–95. doi: 10.1111/ijlh.70217 (PMC13729941; doi:10.1111/ijlh.70217)
Supplement: Supplementary file 1 [file IJLH-48-1385-s001.docx]

**Unsupervised Global CBC/RUO/CPD Phenotyping Identifies Haematological Clusters Enriched for Thrombocytopenia Severity and Mechanisms**

Mohammad A. Altememi¹^,^²*, Emmanuel J. Favaloro¹^,^³, Md Zahid Islam⁴^,^⁵ Sara Ng^6^, Danny Hsu^6^ and Abishek B. Santhakumar¹

**Affiliations**

¹ School of Dentistry and Medical Sciences, Faculty of Science and Health, Charles Sturt University, Wagga Wagga, NSW, Australia

² Haematology Laboratory, Liverpool Hospital, Liverpool, NSW, Australia

³ Sydney Centres for Thrombosis and Haemostasis, Institute of Clinical Pathology and Medical Research (ICPMR) and Research and Education Network (REN), Westmead Hospital, Westmead, NSW, Australia

⁴ AI and Cyber Futures Centre, Charles Sturt University, Bathurst, NSW, Australia

⁵ School of Computing, Mathematics and Engineering, Charles Sturt University, Bathurst, NSW, Australia

^6^ Department of Haematology, Liverpool Hospital, Faculty of Medicine & Health, University of New South Wales.

**ORCID IDs**

Mohammad A. Altememi: <https://orcid.org/0009-0004-2927-6868>; Emmanuel J. Favaloro: <https://orcid.org/0000-0002-2103-1661>; Md Zahid Islam: <https://orcid.org/0000-0002-4868-4945>; Sara Ng <https://orcid.org/0000-0002-5620-1659>; Danny Hsu <https://orcid.org/0000-0002-9577-5401>; Abishek B. Santhakumar: <https://orcid.org/0000-0003-1836-5035>

***Corresponding Author**
Mohammad A. Altememi
Email: maltememi@csu.edu.au

**Figures (main manuscript):** 3

**Tables (main manuscript):** 2

**Supplementary Figures:** 8

**Supplementary Tables:** 8

**Running title: Global CBC/RUO/CPD Phenotyping by Unsupervised ML**

**Key words:** Thrombocytopenia; Unsupervised machine learning; Clustering; Complete blood count; Cell population data

**Acknowledgements:** The authors gratefully acknowledge Sudhir Berry for providing valuable insight into laboratory operational considerations and feasibility aspects of the study. The authors also thank Brooke Porter for assistance with dataset labelling and preparation prior to machine learning analyses and Alana Debono for assistance with data extraction. The authors gratefully acknowledge NSW Health Pathology for providing the valuable data that made this study possible.

**Conflict of interest:** The authors have no conflicts of interest to disclose.

**Data availability:** The data that support the findings of this study are not publicly available due to ethical and privacy restrictions. The dataset contains de-identified patient laboratory data obtained under institutional ethics approval, and release of these data is not permitted under the terms of that approval or applicable privacy regulations.

**Author Contribution(s):** Mohammad A. Altememi conceived and designed the study, performed data processing and machine learning model development, and drafted the manuscript. Emmanuel J. Favaloro and Abishek B. Santhakumar provided domain expertise in hematology and hemostasis, contributed to study design and feasibility considerations, and critically structured and revised the manuscript. Md Zahid Islam provided methodological guidance in machine learning framework development and assisted with model evaluation and validation. Dr Sara Ng and Dr Danny Hsu, Consultant Haematologists, critically reviewed the manuscript, including evaluation of the clinical implications of the findings and contributions from a haematology clinical perspective. All authors critically reviewed multiple drafts of the manuscript, contributed to interpretation of the findings, and approved the final version.

**Funding Information:** This research received no specific grant from any funding agency.

**Supplementary Files**

**Figure S1. PCA cumulative explained variance scree plot.** Cumulative proportion of variance explained by successive principal components derived from the scaled complete-case dataset (123 features; n=173,851). The first principal component accounts for the majority of variance, producing a sharp elbow in the scree curve and indicating that a small number of components capture most of the variability in the full feature space.

**Figure S2. Internal diagnostic plots for selection of cluster number in k-means clustering.** (A) Elbow plot showing k-means inertia (within-cluster sum of squares) across candidate cluster numbers (k = 2–15) in the 20-dimensional PCA feature space, illustrating the diminishing improvement in model fit with increasing k. (B) Silhouette coefficient as a function of cluster number (k), computed using Euclidean distance on a random 10,000-sample subset, showing progressive decline in average cluster separation as k increases. These diagnostic plots were used alongside other internal validation metrics to assess clustering structure and guide selection of an appropriate cluster number.

**Figure S3. k-nearest neighbour distance plot for DBSCAN parameter estimation.** Sorted k-NN distance plot (k = 15) computed in the 20-dimensional PCA feature space using a subsample of 50,000 observations. Distances to the 15^th^ nearest neighbour are plotted in ascending order to identify a potential “knee” in the curve for selection of the DBSCAN ε parameter. Quantile thresholds of the distance distribution are shown for reference (90^th^ ≈ 92.8, 95^th^ ≈ 105.0, 97.5^th^ ≈ 118.0, 99^th^ ≈ 135.9), which were explored as candidate ε values during density-based clustering experiments.

**Figure S4. Sensitivity analysis of thrombocytopenia subsets using k-means clustering (k=5).** Visualization of exploratory clustering using the broader 123-feature analysis performed separately within thrombocytopenia cohorts defined by platelet count thresholds (<150, <100, and <50 ×10⁹/L). For each cohort, numerical CBC and CPD features were standardized and reduced using principal component analysis (PCA), retaining the first 20 principal components (PCA20) prior to clustering. K-means clustering (k=5, k-means++ initialization, n_init=20, random_state=42) was applied within each subset to assess whether additional phenotypic structure emerged under varying thrombocytopenia severity thresholds. (A) PCA projection (PC1 vs PC2) of the PLT <150 ×10⁹/L subset colored by k-means cluster assignments. (B) Two-dimensional t-SNE embedding of the same cohort (subsample visualization) colored by cluster labels. (C) PCA projection (PC1 vs PC2) for the PLT <100 ×10⁹/L subset following PCA20 dimensionality reduction and k-means clustering. (D) Corresponding t-SNE visualization of the PLT <100 cohort colored by cluster membership. (E) PCA projection (PC1 vs PC2) for the PLT <50 ×10⁹/L subset representing severe thrombocytopenia. (F) Corresponding t-SNE embedding showing cluster structure within this severe cohort. Across all severity strata, clusters demonstrated partially separable structure in PCA space with broadly consistent organization in t-SNE embeddings, although cluster dispersion increased as platelet thresholds decreased due to reduced sample size. These analyses were performed as sensitivity checks to evaluate clustering stability across thrombocytopenia severity definitions and were not used for the final phenotypic model reported in the main analysis.

**Figure S5. Exploratory visualization of k-means clustering in the full complete-case PCA feature space.** K-means clustering (k=5) was applied as an exploratory analysis to the full complete-case dataset represented in the 20-dimensional PCA space (n=173,851 specimens). (A) Projection of clusters onto PC1 vs PC2. (B) Overlay of the same cluster assignments onto a t-SNE embedding generated from a 20,000-specimen subsample. (C) Alternate rendering of the PC1 vs PC2 projection with cluster colour scale. (D) Projection of clusters onto PC1 vs PC3. (E) Projection of clusters onto PC2 vs PC3. Across pairwise PCA views, separation was dominated primarily along PC1, whereas substantial overlap persisted in secondary component space and in the t-SNE embedding. These exploratory visualizations support the interpretation that, in the full feature set, clustering structure was driven largely by a dominant variance axis rather than by clearly separated natural groups.

**Figure S6. Exploratory k-means clustering (k=5) in the complete-case PCA space and t-SNE projection.**
(A) K-means clustering with k=5 applied to the complete-case PCA representation of the broader exploratory feature set (n = 173,851), shown in PC1–PC2 space. (B) The same exploratory clustering visualized on a t-SNE embedding derived from a 20,000-case subsample. These analyses were used for exploratory structure assessment and were not the final clustering model used for post hoc clinical annotation/evaluation.

**Figure S7. Sensitivity analysis of the final global k-means clustering solution across thrombocytopenia severity thresholds.** Boxplots show distributions of platelet count (PLT), white blood cell count (WBC; log10[WBC + 0.01]), mean platelet volume (MPV), and plateletcrit (@PCT) across clusters from the final global CBC_EXTENDED k-means model (k=4) after restriction to progressively thrombocytopenic complete-case subsets. The PLT <100 ×10⁹/L subset included 18,077 specimens, with cluster sizes C0=3,120, C1=2,569, C2=1,508, and C3=10,880. The PLT <50 ×10⁹/L subset included 7,562 specimens, with cluster sizes C0=966, C1=748, C2=487, and C3=5,361. Boxes represent interquartile ranges with medians indicated. Cluster 3 was progressively enriched across thrombocytopenia thresholds, supporting its interpretation as the global cytopenic/lower-platelet phenotype within the final k=4 model.

**Figure S8. Distribution of final global k-means clusters across thrombocytopenia severity thresholds.** Stacked bar plots show the proportion of complete-case specimens assigned to each cluster from the final global CBC_EXTENDED k-means model (k=4) across nested platelet count thresholds (PLT <150, <100, and <50 ×10⁹/L). Sample sizes were n=39,333, n=18,077, and n=7,562, respectively. Cluster 3 accounted for 18,147/39,333 (46.14%) PLT <150 cases, 10,880/18,077 (60.19%) PLT <100 cases, and 5,361/7,562 (70.89%) PLT <50 cases, demonstrating progressive enrichment of Cluster 3 with increasing thrombocytopenia severity. These findings show that thrombocytopenic specimens are preferentially represented in Cluster 3 within the final global CBC_EXTENDED k=4 clustering solution.

| **Threshold** | **Cluster** | **CBC_EXTENDED complete-case feature set** | | | | **Broader exploratory complete-case feature set** | | | |
| --- | --- | --- | --- | --- | --- | --- | --- | --- | --- |
|  |  | **n** | **PLT median [IQR]** | **WBC median** | **RBC median** | **n** | **PLT median [IQR]** | **WBC median** | **RBC median** |
| PLT <150 | 0 | 4601 | 40.20 [20.60-86.30] | 1.83 | 2.69 | 1150 | 72.65 [46.52-105.92] | 12.44 | 3.12 |
| PLT <150 | 1 | 8152 | 111.00 [77.07-133.10] | 8.30 | 3.30 | 9132 | 99.90 [66.50-127.10] | 4.54 | 2.97 |
| PLT <150 | 2 | 12803 | 121.80 [93.50-138.40] | 6.49 | 4.30 | 6252 | 110.60 [76.00-132.70] | 9.48 | 3.39 |
| PLT <150 | 3 | 13640 | 95.80 [56.30-125.60] | 4.37 | 3.09 | 8933 | 124.60 [99.90-139.30] | 6.05 | 4.31 |
| PLT <150 | 4 | 137 | 59.80 [42.20-98.50] | 199.18 | 3.01 | 4158 | 38.85 [20.90-72.88] | 2.16 | 2.86 |
| PLT <100 | 0 | 2636 | 26.50 [17.00-47.30] | 0.86 | 2.68 | 1749 | 30.20 [18.00-52.00] | 1.47 | 2.78 |
| PLT <100 | 1 | 6184 | 54.20 [28.50-78.00] | 3.15 | 2.89 | 3897 | 75.10 [53.10-89.20] | 6.31 | 4.02 |
| PLT <100 | 2 | 4985 | 72.10 [47.30-88.20] | 5.94 | 4.01 | 2327 | 36.60 [21.70-62.60] | 3.19 | 2.90 |
| PLT <100 | 3 | 61 | 51.10 [38.80-60.00] | 327.38 | 2.94 | 4956 | 67.40 [45.50-84.80] | 4.24 | 2.85 |
| PLT <100 | 4 | 4211 | 66.30 [41.10-84.80] | 6.88 | 2.94 | 803 | 58.00 [37.90-77.10] | 14.04 | 3.04 |
| PLT <50 | 0 | 50 | 34.25 [28.75-42.20] | 218.96 | 2.79 | 1148 | 24.70 [16.30-33.23] | 2.15 | 2.80 |
| PLT <50 | 1 | 1437 | 21.30 [15.10-30.30] | 0.62 | 2.69 | 1366 | 33.50 [19.30-42.40] | 6.39 | 3.77 |
| PLT <50 | 2 | 3025 | 26.20 [17.30-37.20] | 2.04 | 2.77 | 921 | 22.50 [15.50-32.00] | 1.10 | 2.74 |
| PLT <50 | 3 | 2285 | 31.10 [19.00-41.00] | 5.99 | 3.42 | 384 | 31.30 [20.10-41.42] | 12.08 | 2.89 |
| PLT <50 | 4 | 765 | 24.10 [16.10-35.60] | 0.77 | 2.69 | 1593 | 28.80 [18.90-39.30] | 2.38 | 2.73 |

**Table S1. Generated thrombocytopenia restricted exploratory k-means sensitivity analyses across two feature spaces.** K-means clustering (k=5) was performed separately within nested platelet defined thrombocytopenia subsets (PLT <150, <100, and <50 ×10⁹/L) using two independently fitted feature spaces: the CBC_EXTENDED 41-feature complete-case feature set used in the primary global modelling workflow, and a broader missingness-filtered 123-feature exploratory complete-case feature set used for supplementary sensitivity analysis. Within each platelet defined subset and feature space, variables were scaled, reduced using principal component analysis with 20 components, and clustered using k-means++ initialisation with n_init=20 and random_state=42. CBC_EXTENDED complete-case subset sizes were 39,333 for PLT <150 ×10⁹/L, 18,077 for PLT <100 ×10⁹/L, and 7,562 for PLT <50 ×10⁹/L. The corresponding broader exploratory complete-case subset sizes were 29,625, 13,732, and 5,412, respectively. Values are shown as cluster size, platelet median [25th–75th percentile], WBC median, and RBC median. Because clustering was fitted independently within each feature space and platelet threshold, cluster numbers are model specific and should not be interpreted as matched across feature spaces, platelet thresholds, or the final global k=4 model. These analyses were exploratory sensitivity assessments and were not used to select, train, validate, or interpret the final global k=4 CBC_EXTENDED model.

| **Item** | **CORE_CBC** | **CBC_EXTENDED** | **FULL_MODEL** |
| --- | --- | --- | --- |
| **Complete-case retention** | | | |
| Number of features | 6 | 41 | 173 |
| Total specimens | 254,289 | 254,289 | 254,289 |
| Complete cases retained | 253,384 | 241,591 | 0 |
| Specimens excluded | 905 | 12,698 | 254,289 |
| Retained fraction | 0.9964 | 0.9501 | 0.0000 |
| Retained percentage | 99.64% | 95.01% | 0.00% |
| **Exact features included in each feature set** | | | |
| Feature 001 | PLT | WBC | Age |
| Feature 002 | WBC | UWBC | WBC |
| Feature 003 | RBC | RBC | UWBC |
| Feature 004 | HGB | HGB | RBC |
| Feature 005 | MCV | MCV | HGB |
| Feature 006 | RDW | MCH | HCT |
| Feature 007 |  | MCHC | MCV |
| Feature 008 |  | RDW | MCH |
| Feature 009 |  | RDW-SD | MCHC |
| Feature 010 |  | PLT | RDW |
| Feature 011 |  | MPV | RDW-SD |
| Feature 012 |  | NRBC | PLT |
| Feature 013 |  | NRBC# | MPV |
| Feature 014 |  | @MN-V-NRBC | NE |
| Feature 015 |  | @SD-V-NRBC | LY |
| Feature 016 |  | @MN-C-NRBC | MO |
| Feature 017 |  | @SD-C-NRBC | EO |
| Feature 018 |  | @MN-MALS-NRBC | BA |
| Feature 019 |  | @SD-MALS-NRBC | NE# |
| Feature 020 |  | @MN-UMALS-NRBC | NRBC |
| Feature 021 |  | @SD-UMALS-NRBC | NRBC# |
| Feature 022 |  | @MN-LMALS-NRBC | RET |
| Feature 023 |  | @SD-LMALS-NRBC | RET# |
| Feature 024 |  | @MN-LALS-NRBC | MRV |
| Feature 025 |  | @SD-LALS-NRBC | IRF |
| Feature 026 |  | @MN-AL2-NRBC | TNC |
| Feature 027 |  | @SD-AL2-NRBC | @PCT |
| Feature 028 |  | @MN-V-NNRBC | @PDW |
| Feature 029 |  | @SD-V-NNRBC | @WDOP |
| Feature 030 |  | @MN-C-NNRBC | @WNOP |
| Feature 031 |  | @SD-C-NNRBC | @UGC# |
| Feature 032 |  | @MN-MALS-NNRBC | @WROP |
| Feature 033 |  | @SD-MALS-NNRBC | @UWROP |
| Feature 034 |  | @MN-UMALS-NNRBC | @MN-V-NE |
| Feature 035 |  | @SD-UMALS-NNRBC | @SD-V-NE |
| Feature 036 |  | @MN-LMALS-NNRBC | @MN-C-NE |
| Feature 037 |  | @SD-LMALS-NNRBC | @SD-C-NE |
| Feature 038 |  | @MN-LALS-NNRBC | @MN-MALS-NE |
| Feature 039 |  | @SD-LALS-NNRBC | @SD-MALS-NE |
| Feature 040 |  | @MN-AL2-NNRBC | @MN-UMALS-NE |
| Feature 041 |  | @SD-AL2-NNRBC | @SD-UMALS-NE |
| Feature 042 |  |  | @MN-LMALS-NE |
| Feature 043 |  |  | @SD-LMALS-NE |
| Feature 044 |  |  | @MN-LALS-NE |
| Feature 045 |  |  | @SD-LALS-NE |
| Feature 046 |  |  | @MN-AL2-NE |
| Feature 047 |  |  | @SD-AL2-NE |
| Feature 048 |  |  | @MN-V-LY |
| Feature 049 |  |  | @SD-V-LY |
| Feature 050 |  |  | @MN-C-LY |
| Feature 051 |  |  | @SD-C-LY |
| Feature 052 |  |  | @MN-MALS-LY |
| Feature 053 |  |  | @SD-MALS-LY |
| Feature 054 |  |  | @MN-UMALS-LY |
| Feature 055 |  |  | @SD-UMALS-LY |
| Feature 056 |  |  | @MN-LMALS-LY |
| Feature 057 |  |  | @SD-LMALS-LY |
| Feature 058 |  |  | @MN-LALS-LY |
| Feature 059 |  |  | @SD-LALS-LY |
| Feature 060 |  |  | @MN-AL2-LY |
| Feature 061 |  |  | @SD-AL2-LY |
| Feature 062 |  |  | @MN-V-MO |
| Feature 063 |  |  | @SD-V-MO |
| Feature 064 |  |  | @MN-C-MO |
| Feature 065 |  |  | @SD-C-MO |
| Feature 066 |  |  | @MN-MALS-MO |
| Feature 067 |  |  | @SD-MALS-MO |
| Feature 068 |  |  | @MN-UMALS-MO |
| Feature 069 |  |  | @SD-UMALS-MO |
| Feature 070 |  |  | @MN-LMALS-MO |
| Feature 071 |  |  | @SD-LMALS-MO |
| Feature 072 |  |  | @MN-LALS-MO |
| Feature 073 |  |  | @SD-LALS-MO |
| Feature 074 |  |  | @MN-AL2-MO |
| Feature 075 |  |  | @SD-AL2-MO |
| Feature 076 |  |  | @MN-V-EO |
| Feature 077 |  |  | @SD-V-EO |
| Feature 078 |  |  | @MN-C-EO |
| Feature 079 |  |  | @SD-C-EO |
| Feature 080 |  |  | @MN-MALS-EO |
| Feature 081 |  |  | @SD-MALS-EO |
| Feature 082 |  |  | @MN-UMALS-EO |
| Feature 083 |  |  | @SD-UMALS-EO |
| Feature 084 |  |  | @MN-LMALS-EO |
| Feature 085 |  |  | @SD-LMALS-EO |
| Feature 086 |  |  | @MN-LALS-EO |
| Feature 087 |  |  | @SD-LALS-EO |
| Feature 088 |  |  | @MN-AL2-EO |
| Feature 089 |  |  | @SD-AL2-EO |
| Feature 090 |  |  | @MN-V-EGC |
| Feature 091 |  |  | @SD-V-EGC |
| Feature 092 |  |  | @MN-C-EGC |
| Feature 093 |  |  | @SD-C-EGC |
| Feature 094 |  |  | @MN-MALS-EGC |
| Feature 095 |  |  | @SD-MALS-EGC |
| Feature 096 |  |  | @MN-UMALS-EGC |
| Feature 097 |  |  | @SD-UMALS-EGC |
| Feature 098 |  |  | @MN-LMALS-EGC |
| Feature 099 |  |  | @SD-LMALS-EGC |
| Feature 100 |  |  | @MN-LALS-EGC |
| Feature 101 |  |  | @SD-LALS-EGC |
| Feature 102 |  |  | @MN-AL2-EGC |
| Feature 103 |  |  | @SD-AL2-EGC |
| Feature 104 |  |  | @MN-V-NRBC |
| Feature 105 |  |  | @SD-V-NRBC |
| Feature 106 |  |  | @MN-C-NRBC |
| Feature 107 |  |  | @SD-C-NRBC |
| Feature 108 |  |  | @MN-MALS-NRBC |
| Feature 109 |  |  | @SD-MALS-NRBC |
| Feature 110 |  |  | @MN-UMALS-NRBC |
| Feature 111 |  |  | @SD-UMALS-NRBC |
| Feature 112 |  |  | @MN-LMALS-NRBC |
| Feature 113 |  |  | @SD-LMALS-NRBC |
| Feature 114 |  |  | @MN-LALS-NRBC |
| Feature 115 |  |  | @SD-LALS-NRBC |
| Feature 116 |  |  | @MN-AL2-NRBC |
| Feature 117 |  |  | @SD-AL2-NRBC |
| Feature 118 |  |  | @MN-V-NNRBC |
| Feature 119 |  |  | @SD-V-NNRBC |
| Feature 120 |  |  | @MN-C-NNRBC |
| Feature 121 |  |  | @SD-C-NNRBC |
| Feature 122 |  |  | @MN-MALS-NNRBC |
| Feature 123 |  |  | @SD-MALS-NNRBC |
| Feature 124 |  |  | @MN-UMALS-NNRBC |
| Feature 125 |  |  | @SD-UMALS-NNRBC |
| Feature 126 |  |  | @MN-LMALS-NNRBC |
| Feature 127 |  |  | @SD-LMALS-NNRBC |
| Feature 128 |  |  | @MN-LALS-NNRBC |
| Feature 129 |  |  | @SD-LALS-NNRBC |
| Feature 130 |  |  | @MN-AL2-NNRBC |
| Feature 131 |  |  | @SD-AL2-NNRBC |
| Feature 132 |  |  | @MN-V-UGC |
| Feature 133 |  |  | @SD-V-UGC |
| Feature 134 |  |  | @MN-C-UGC |
| Feature 135 |  |  | @SD-C-UGC |
| Feature 136 |  |  | @MN-MALS-UGC |
| Feature 137 |  |  | @SD-MALS-UGC |
| Feature 138 |  |  | @MN-UMALS-UGC |
| Feature 139 |  |  | @SD-UMALS-UGC |
| Feature 140 |  |  | @MN-LMALS-UGC |
| Feature 141 |  |  | @SD-LMALS-UGC |
| Feature 142 |  |  | @MN-LALS-UGC |
| Feature 143 |  |  | @SD-LALS-UGC |
| Feature 144 |  |  | @MN-AL2-UGC |
| Feature 145 |  |  | @SD-AL2-UGC |
| Feature 146 |  |  | @MN-V-RET |
| Feature 147 |  |  | @SD-V-RET |
| Feature 148 |  |  | @MN-C-RET |
| Feature 149 |  |  | @SD-C-RET |
| Feature 150 |  |  | @MN-MALS-RET |
| Feature 151 |  |  | @SD-MALS-RET |
| Feature 152 |  |  | @MN-UMALS-RET |
| Feature 153 |  |  | @SD-UMALS-RET |
| Feature 154 |  |  | @MN-LMALS-RET |
| Feature 155 |  |  | @SD-LMALS-RET |
| Feature 156 |  |  | @MN-LALS-RET |
| Feature 157 |  |  | @SD-LALS-RET |
| Feature 158 |  |  | @MN-AL2-RET |
| Feature 159 |  |  | @SD-AL2-RET |
| Feature 160 |  |  | @MN-V-NRET |
| Feature 161 |  |  | @SD-V-NRET |
| Feature 162 |  |  | @MN-C-NRET |
| Feature 163 |  |  | @SD-C-NRET |
| Feature 164 |  |  | @MN-MALS-NRET |
| Feature 165 |  |  | @SD-MALS-NRET |
| Feature 166 |  |  | @MN-UMALS-NRET |
| Feature 167 |  |  | @SD-UMALS-NRET |
| Feature 168 |  |  | @MN-LMALS-NRET |
| Feature 169 |  |  | @SD-LMALS-NRET |
| Feature 170 |  |  | @MN-LALS-NRET |
| Feature 171 |  |  | @SD-LALS-NRET |
| Feature 172 |  |  | @MN-AL2-NRET |
| Feature 173 |  |  | @SD-AL2-NRET |
| **Complete-case bias analysis for key CBC variables** | | | |
| PLT: retained median | 235.5 | 235.8 | NA |
| PLT: excluded median | 3.4 | 224.9 | 235.3 |
| PLT: Mann-Whitney P | 2.36E-131 | 2.39E-39 | NA |
| WBC: retained median | 7.636 | 7.6 | NA |
| WBC: excluded median | 0.02 | 8.44 | 7.63 |
| WBC: Mann-Whitney P | 4.18E-121 | 1.50E-52 | NA |
| RBC: retained median | 4.089 | 4.101 | NA |
| RBC: excluded median | 0.003 | 3.766 | 4.085 |
| RBC: Mann-Whitney P | <1.0E-300 | <1.0E-300 | NA |
| HGB: retained median | 120.1 | 120.4 | NA |
| HGB: excluded median | 0.5 | 112.1 | 120.0 |
| HGB: Mann-Whitney P | <1.0E-300 | 1.18E-267 | NA |
| MCV: retained median | 89.51 | 89.49 | NA |
| MCV: excluded median | 154.8 | 90.02 | 89.52 |
| MCV: Mann-Whitney P | 1.39E-41 | 7.02E-31 | NA |
| RDW: retained median | 14.65 | 14.65 | NA |
| RDW: excluded median | 41.47 | 14.71 | 14.65 |
| RDW: Mann-Whitney P | 9.92E-24 | 8.57E-06 | NA |

**Table S2. Candidate feature-set definitions, complete-case retention and complete-case bias analysis**. Complete-case retention was evaluated across three candidate feature sets constructed from 254,289 specimen-level records: CORE_CBC, comprising six standard CBC parameters; CBC_EXTENDED, comprising 41 CBC/RUO/CPD-related parameters used for the primary global clustering model; and FULL_MODEL, comprising 173 numeric parameters after exclusion of non-analytic identifiers and date/time fields. CORE_CBC retained 253,384 specimens (99.64%), CBC_EXTENDED retained 241,591 specimens (95.01%), and FULL_MODEL yielded no complete cases because of pervasive missingness across research parameters. To assess potential selection bias from complete-case filtering, retained and excluded specimens were compared for PLT, WBC, RBC, HGB, MCV and RDW using medians and Mann–Whitney U tests. Although statistically significant differences were observed, reflecting the large sample size, absolute differences for the CBC_EXTENDED feature set were small and not suggestive of clinically meaningful distortion. For FULL_MODEL, retained-case medians and Mann–Whitney U p-values were not applicable.

| **Method** | **k** | **Silhouette_Sampled** | **Davies_Bouldin** | **Calinski_Harabasz** |
| --- | --- | --- | --- | --- |
| KMeans | 2 | 0.147403 | 2.275875 | 38395.27 |
| KMeans | 3 | 0.114168 | 2.293422 | 30498.91 |
| KMeans | 4 | 0.098641 | 2.21647 | 25878.52 |
| KMeans | 5 | 0.099888 | 1.917616 | 22480.52 |
| KMeans | 6 | 0.078424 | 2.181509 | 20570.48 |
| KMeans | 7 | 0.073992 | 2.113985 | 18664.77 |
| KMeans | 8 | 0.073362 | 2.191221 | 17452.75 |
| KMeans | 9 | 0.075346 | 1.893963 | 17124.79 |
| KMeans | 10 | 0.075495 | 1.933336 | 16293.44 |

**Table S3. Internal validation metrics for k-means cluster-number assessment in the full PCA20 cohort.** K-means models were fitted across k=2–10 clusters in the 20-dimensional PCA feature space of the full CBC_EXTENDED complete-case cohort (n=241,591). The silhouette coefficient was computed on a fixed random subset of 10,000 specimens for computational feasibility, whereas the Davies–Bouldin index, Calinski–Harabasz index and model inertia were computed on the full PCA20 cohort. These metrics were used as internal diagnostics to compare candidate k values and guide selection of an interpretable clustering solution.

| **Parameter** | **Cluster 0 median [IQR]** | **Cluster 1 median [IQR]** | **Cluster 2 median [IQR]** | **Cluster 3 median [IQR]** | **Kruskal-Wallis p** | **ε²** |
| --- | --- | --- | --- | --- | --- | --- |
| **BA** | 0.57 [0.38-0.8] | 0.37 [0.21-0.59] | 0.37 [0.21-0.6] | 0.53 [0.3-0.84] | <1.0E-300 | 0.0678 |
| **EO** | 2.16 [1.14-3.64] | 0.89 [0.22-2.13] | 1.11 [0.27-2.51] | 1.7 [0.55-3.47] | <1.0E-300 | 0.0811 |
| **HCT** | 0.39 [0.35-0.42] | 0.4 [0.36-0.44] | 0.35 [0.31-0.39] | 0.28 [0.25-0.31] | <1.0E-300 | 0.4342 |
| **HGB** | 128 [116.8-139.2] | 133.4 [120.1-146.7] | 116 [101.5-129.2] | 92.5 [83.8-102.4] | <1.0E-300 | 0.4434 |
| **IRF** | 41.8 [35-49.7] | 47.8 [38.4-58.7] | 42.5 [33.3-51.2] | 43.95 [26.9-56.4] | 1.21E-88 | 0.0193 |
| **LY** | 28.11 [22.4-34.66] | 13.16 [8.54-18.02] | 14.16 [8.71-20.45] | 20.02 [12.56-30.83] | <1.0E-300 | 0.3444 |
| **MCH** | 29.79 [28.22-31.16] | 30 [28.53-31.4] | 29.81 [28.17-31.19] | 29.83 [27.59-31.65] | 3.86E-108 | 0.0021 |
| **MCHC** | 333.2 [327.5-338.5] | 333.1 [326.9-338.7] | 332.2 [326.1-337.9] | 329.5 [321.2-337.1] | <1.0E-300 | 0.0191 |
| **MCV** | 89.1 [85.11-92.9] | 89.83 [86.07-93.77] | 89.36 [85.39-93.08] | 89.91 [84.57-95.04] | 1.34E-200 | 0.0038 |
| **MO** | 8.37 [6.77-10.36] | 7.44 [5.6-9.58] | 7.63 [5.84-9.69] | 8.96 [6.58-11.84] | <1.0E-300 | 0.031 |
| **MPV** | 8.41 [7.77-9.14] | 8.51 [7.85-9.25] | 8.27 [7.62-9.01] | 8.26 [7.58-9] | <1.0E-300 | 0.0086 |
| **NE** | 59.38 [52.21-65.86] | 76.65 [70.43-83.17] | 75.07 [67.32-82.37] | 65.76 [53.3-75.34] | <1.0E-300 | 0.3287 |
| **NE#** | 3.97 [2.96-5.23] | 7.18 [5.29-9.8] | 6.88 [4.92-9.67] | 4.03 [2.47-6.02] | <1.0E-300 | 0.258 |
| **NRBC** | 0.1 [0.06-0.15] | 0.08 [0.05-0.13] | 0.02 [0.01-0.04] | 0.11 [0.06-0.24] | <1.0E-300 | 0.236 |
| **NRBC#** | 0.01 [0-0.01] | 0.01 [0.01-0.01] | 0 [0-0] | 0.01 [0-0.01] | <1.0E-300 | 0.1574 |
| **PLT** | 241.1 [191.4-299.7] | 236.4 [183.9-300.6] | 249.1 [189.6-325.4] | 210.9 [128.1-304.1] | <1.0E-300 | 0.0171 |
| **RBC** | 4.36 [3.94-4.77] | 4.49 [4.02-4.96] | 3.95 [3.45-4.42] | 3.15 [2.79-3.58] | <1.0E-300 | 0.3631 |
| **RDW** | 14.14 [13.4-15.31] | 14.35 [13.52-15.77] | 14.45 [13.57-15.86] | 16.67 [14.96-19.08] | <1.0E-300 | 0.1668 |
| **RDW-SD** | 43.75 [41.13-47.69] | 45.06 [42-49.88] | 45.06 [42-49.44] | 51.63 [46.38-59.5] | <1.0E-300 | 0.1539 |
| **RET** | 1.34 [0.96-1.91] | 1.7 [1.11-2.92] | 1.36 [0.86-2.1] | 1.58 [0.59-3.02] | 5.85E-60 | 0.013 |
| **RET#** | 57.75 [40.95-80.57] | 72.77 [46.5-124.4] | 51.72 [30.81-77.95] | 47.55 [16.73-91.84] | 8.71E-194 | 0.0423 |
| **UWBC** | 6.85 [5.43-8.54] | 9.55 [7.35-12.41] | 9.37 [7.17-12.28] | 6.36 [4.45-8.77] | <1.0E-300 | 0.1597 |
| **WBC** | 6.84 [5.42-8.53] | 9.54 [7.35-12.38] | 9.37 [7.17-12.27] | 6.34 [4.44-8.74] | <1.0E-300 | 0.1609 |

**Table S4. Cluster-wise distributions of CBC parameters across the final global k-means model (k=4).** Median values and interquartile ranges (IQRs) are shown for 23 CBC parameters across the four clusters identified by the final global CBC_EXTENDED k-means model. The model was fitted in PCA20 feature space using 241,591 complete-case specimens. Cluster sizes were 89,364 for cluster 0, 58,618 for cluster 1, 34,858 for cluster 2 and 58,751 for cluster 3. Overall, between-cluster differences were assessed using Kruskal–Wallis tests based on available non-missing values for each parameter, with epsilon-squared (ε²) reported as a non-parametric effect-size measure. These summaries were used to define the global cluster phenotypes before assessing post hoc thrombocytopenia and clinical-mechanism enrichment.

| **Feature** | **Cluster 0, n=70** | **Cluster 1, n=54** | **Cluster 2, n=33** | **Cluster 3, n=68** | **Kruskal-Wallis** **H** | **Raw** ***p*** | **BH-adjusted** ***p*** | **Epsilon-squared** **(ε²)** |
| --- | --- | --- | --- | --- | --- | --- | --- | --- |
| **PLT** | 85.90 [43.55-115.83] | 78.25 [45.55-115.18] | 75.90 [58.30-113.30] | 81.40 [29.73-112.68] | 1.99 | 0.575 | 0.575 | 0.000 |
| **WCC** | 4.61 [3.11-6.12] | 8.07 [4.09-11.24] | 7.29 [3.93-12.08] | 3.71 [1.56-5.89] | 31.27 | <0.001 | <0.001 | 0.128 |
| **MPV** | 9.54 [8.50-10.19] | 9.14 [8.71-9.82] | 9.24 [8.43-10.06] | 8.61 [7.88-9.50] | 13.20 | 0.004 | 0.008 | 0.046 |
| **@PCT** | 0.08 [0.04-0.11] | 0.07 [0.04-0.11] | 0.07 [0.05-0.12] | 0.07 [0.03-0.10] | 4.06 | 0.255 | 0.339 | 0.005 |

**Table S5. Cluster-wise haematological profiles in the post hoc clinical annotation cohort.** Values are median [interquartile range] for selected complete blood count and platelet parameters across the four clusters in the 225-specimen post hoc clinical annotation cohort mapped to the final global k = 4 solution. PLT, platelet count; WCC, white cell count; MPV, mean platelet volume; @PCT, plateletcrit. Overall, between-cluster differences were assessed using Kruskal-Wallis tests. Raw and Benjamini-Hochberg (BH)-adjusted p values are reported, with BH adjustment applied across the four tested features; epsilon-squared (ε²) is the omnibus effect-size estimate. Patient-level independence was not established. No pairwise comparisons were undertaken. Cluster assignments should be interpreted as post hoc phenotypic annotations.

| **Model** | **n** | **Purity** | **ARI** | **NMI** |
| --- | --- | --- | --- | --- |
| **KMeans_k4** | 225 | 0.59 | 0.14 | 0.18 |
| **GMM_k3** | 225 | 0.52 | 0.08 | 0.08 |
| **GMM_k4** | 225 | 0.49 | 0.05 | 0.09 |

**Table S6.** **Post-hoc clinical annotation/evaluation** **metrics for unsupervised clustering models.** Performance metrics comparing unsupervised clustering assignments with clinically adjudicated thrombocytopenia mechanisms in the post-hoc clinical annotation/evaluation cohort (n = 225). Agreement between model-derived clusters and EHR-derived mechanism labels was assessed using cluster purity, adjusted Rand index (ARI), and normalized mutual information (NMI). Among models compared post hoc against adjudicated mechanism labels, k-means k=4 showed the highest concordance, although agreement remained limited. These findings indicate partial clinical enrichment of the selected global k=4 solution rather than external validation or diagnostic classification.

| **Model** | **k** | **Analysis** | **n_eval** | **Purity** | **ARI** | **NMI** | **Purity_rank** | **ARI_rank** | **NMI_rank** |
| --- | --- | --- | --- | --- | --- | --- | --- | --- | --- |
| **GMM** | 3 | ALL | 225 | 0.52 | 0.08 | 0.08 | 3 | 3 | 4 |
| **GMM** | 4 | ALL | 225 | 0.49 | 0.05 | 0.09 | 4 | 4 | 3 |
| **KMeans** | 4 | ALL | 225 | 0.59 | 0.15 | 0.18 | 2 | 1 | 2 |
| **Spectral** | 4 | SUBSAMPLE | 13 | 0.69 | 0.09 | 0.23 | 1 | 2 | 1 |

**Table S7. Comparative summary of clustering model performance against clinically adjudicated thrombocytopenia mechanisms.** Comparison of unsupervised clustering approaches evaluated against EHR-derived thrombocytopenia mechanism labels in the post-hoc clinical annotation/evaluation cohort. Agreement between model-derived clusters and clinical labels was assessed using cluster purity, adjusted Rand index (ARI), and normalized mutual information (NMI). Gaussian mixture models (GMM; k=3 and k=4) and k-means (k=4) were fitted in the PCA20 feature space of the full dataset and evaluated on the 225 cases. An exploratory spectral clustering model (k=4) was applied to a 12,000-specimen subsample, resulting in overlap with only 13 enriched cases, and is therefore presented for qualitative comparison only. Across models evaluated on the full post hoc clinical annotation cohort, k-means (k=4) demonstrated the strongest concordance with clinical labels (purity = 0.59, ARI = 0.15, NMI = 0.18). Rank columns indicate relative model performance across the three evaluation metrics.

| **Model** | **Labelled cases** | **ARI** | **AMI** | **V-measure** | **χ² p-value** | **Cramér’s V** | **Interpretation** |
| --- | --- | --- | --- | --- | --- | --- | --- |
| **KMeans (k=4)** | 225 | 0.144 | 0.166 | 0.18 | 9.0 × 10⁻¹⁹ | 0.397 | Moderate concordance |
| **GMM (k=3)** | 225 | 0.077 | 0.073 | 0.085 | 5.6 × 10⁻⁷ | 0.296 | Weak concordance |
| **GMM (k=4)** | 225 | 0.054 | 0.069 | 0.085 | 4.2 × 10⁻⁶ | 0.248 | Weak concordance |

**Table S8. Concordance and statistical association between clustering models and clinically adjudicated thrombocytopenia mechanisms.** Agreement between unsupervised clustering assignments and clinically adjudicated thrombocytopenia mechanisms in the post-hoc clinical annotation/evaluation cohort (n = 225) was evaluated using multiple complementary metrics, including the adjusted Rand index (ARI), adjusted mutual information (AMI), and V-measure. Statistical association between cluster membership and mechanism labels was additionally assessed using the chi-square test of independence and Cramér’s V as a measure of effect size. Across models evaluated on the full PCA20 feature space, the k-means k=4 model demonstrated the strongest concordance with clinical labels, with moderate association strength (Cramér’s V = 0.397) and highly significant association (χ² p = 9.0 × 10⁻¹⁹), whereas Gaussian mixture models showed weaker concordance. Noise fraction represents the proportion of enriched cases assigned to clusters inconsistent with their clinically adjudicated mechanism.
